# Supplementary material for: Transposable Elements Drive Regulatory and Functional Innovation of F-box Genes
Source: Mol Biol Evol. 2025 Apr 25;42(5):msaf097. doi: 10.1093/molbev/msaf097 (PMC12062965; doi:10.1093/molbev/msaf097)
Supplement: msaf097_Supplementary_Data [file msaf097_supplementary_data.zip › Reference.docx]

Robinson JT, Thorvaldsdóttir H, Winckler W, Guttman M, Lander ES, Getz G, Mesirov JP. 2011. Integrative genomics viewer. *Nat Biotechnol*. 29:24-26.
